# Brain recognition of previously learned versus novel temporal sequences: a differential simultaneous processing

**DOI:** 10.1093/cercor/bhac439

**Published:** 2022-11-08

**Authors:** L Bonetti, E Brattico, S E P Bruzzone, G Donati, G Deco, D Pantazis, P Vuust, M L Kringelbach

**Affiliations:** Center for Music in the Brain (MIB), Department of Clinical Medicine, Aarhus University & The Royal Academy of Music Aarhus/Aalborg, Universitetsbyen 3, 8000, Aarhus C, Denmark; Centre for Eudaimonia and Human Flourishing, Linacre College, University of Oxford, Stoke place 7, OX39BX, Oxford, UK; Department of Psychiatry, University of Oxford, Oxford, UK; Department of Psychology, University of Bologna, Italy; Center for Music in the Brain (MIB), Department of Clinical Medicine, Aarhus University & The Royal Academy of Music Aarhus/Aalborg, Universitetsbyen 3, 8000, Aarhus C, Denmark; Department of Education, Psychology, Communication, University of Bari Aldo Moro, Italy; Center for Music in the Brain (MIB), Department of Clinical Medicine, Aarhus University & The Royal Academy of Music Aarhus/Aalborg, Universitetsbyen 3, 8000, Aarhus C, Denmark; Neurobiology Research Unit (NRU), Copenhagen University Hospital Rigshospitalet, Inge Lehmanns Vej 6, 2100, Copenhagen, Denmark; Faculty of Health and Medical Sciences, University of Copenhagen, Blegdamsvej 3B, 2200, Copenhagen, Denmark; Department of Psychology, University of Bologna, Italy; Computational and Theoretical Neuroscience Group, Center for Brain and Cognition, Universitat Pompeu Fabra, Edifici Merce Rodereda, C/ de Ramon Trias Fargas, 25, 08018 Barcelona, Spain; McGovern Institute for Brain Research, Massachusetts Institute of Technology (MIT), 77 Massachusetts Ave, Cambridge, MA 02139, USA; Center for Music in the Brain (MIB), Department of Clinical Medicine, Aarhus University & The Royal Academy of Music Aarhus/Aalborg, Universitetsbyen 3, 8000, Aarhus C, Denmark; Center for Music in the Brain (MIB), Department of Clinical Medicine, Aarhus University & The Royal Academy of Music Aarhus/Aalborg, Universitetsbyen 3, 8000, Aarhus C, Denmark; Centre for Eudaimonia and Human Flourishing, Linacre College, University of Oxford, Stoke place 7, OX39BX, Oxford, UK; Department of Psychiatry, University of Oxford, Oxford, UK

**Keywords:** temporal sequences, memory recognition, magnetoencephalography, brain dynamics, source reconstruction

## Abstract

Memory for sequences is a central topic in neuroscience, and decades of studies have investigated the neural mechanisms underlying the coding of a wide array of sequences extended over time. Yet, little is known on the brain mechanisms underlying the recognition of previously memorized versus novel temporal sequences. Moreover, the differential brain processing of single items in an auditory temporal sequence compared to the whole superordinate sequence is not fully understood. In this magnetoencephalography (MEG) study, the items of the temporal sequence were independently linked to local and rapid (2–8 Hz) brain processing, while the whole sequence was associated with concurrent global and slower (0.1–1 Hz) processing involving a widespread network of sequentially active brain regions. Notably, the recognition of previously memorized temporal sequences was associated to stronger activity in the slow brain processing, while the novel sequences required a greater involvement of the faster brain processing. Overall, the results expand on well-known information flow from lower- to higher order brain regions. In fact, they reveal the differential involvement of slow and faster whole brain processing to recognize previously learned versus novel temporal information.

## Introduction

Over the years, understanding memory for sequences has been a central topic in neuroscience and cognitive science (Förstl et al. 2006; Kumar and Anand 2015), as reviewed in a key paper by Dehaene et al. (2015). Here, the authors propose a taxonomy of the processing mechanisms for temporal sequences, arranged in five categories of increasing complexity: (i) transition and timing knowledge between subsequent items of the sequence, (ii) chunking of contiguous items of the sequence, (iii) ordinal knowledge of which item comes first, (iv) algebraic patterns capturing complex regularities within a sequence, and (v) nested tree structures based on abstract symbolic rules.

The first category refers to the processing of the transition from one item of the sequence to the next. This research has identified several automatic event related potentials/fields (ERP/F) to standard and deviant sounds, such as the well-known N100 and mismatch negativity (MMN), demonstrating that the brain can rapidly detect changes in the regularity of sequences (Garrido et al. 2009; Lijffijt et al. 2009; Vuust et al. 2012; Bonetti et al. 2017, 2018; Bonetti, Brattico, Carlomagno, et al. 2021a; Bonetti, Bruzzone, Sedghi, et al. 2021b; Bonetti, Brattico, Vuust, et al. 2021c).

Chunking refers to the ability of grouping contiguous items into a larger single unit.

Previous studies at the single-cell level have shown that chunking occurs during acquisition of motor habits (Fujii and Graybiel 2003; Smith and Graybiel 2013; Jin et al. 2014).

Similar studies with functional magnetic resonance imaging (fMRI) confirmed these results, suggesting a hierarchical organization in chunking when motor temporal sequences are learned (Koechlin and Jubault 2006). The neural correlates of auditory chunking were further investigated by additional studies. For instance, Kalm et al. (2012) used fMRI to explore the auditory encoding of grouped and ungrouped lists of letters, highlighting that a large activation of auditory cortex, premotor and prefrontal brain areas was associated with exceeded memory span during encoding. Additionally, using electroencephalography (EEG), Ding et al. (2018) discovered that neural encoding of individual auditory events (i.e. syllables) was automatic, while knowledge-based construction of temporal chunks (i.e. words) relied on attention.

Beyond chunking, ordinal knowledge describes the capacity of learning and remembering which item comes first, second, and so on, in the temporal sequence. Previous studies have demonstrated that the ordinal arrangement of a series of items is encoded by the brain within the intraparietal and dorsolateral prefrontal cortices (Terrace et al. 2003; Berdyyeva and Olson 2010; Crowe et al. 2014).

Algebraic patterns refer to the abstract schemas stored in the brain, which enable the capture of sequential regularities underlying a sequence of items. Previous research suggested that the brain processes the algebraic patterns of sequences, understanding a higher level of abstract regularities than elementary sequences with sensorial deviations (Endress et al. 2009; Wang et al. 2015).

Finally, nested tree structures are generated by symbolic rules, which are often embedded within each other. Previous studies have highlighted the following network of brain regions involved in processing complex linguistic patterns organized in nested structures: left superior temporal sulcus (STS), middle temporal gyrus (MTG), temporal poles (TP) inferior frontal gyrus (IFG), and temporo-parietal junction (TPJ) (Mazoyer et al. 1993; Saur et al. 2010; Friederici 2011; Rolheiser et al. 2011; Tyler et al. 2011).

Other relevant contributions on how the brain processes temporal sequences came from research in auditory and speech processing. This proposed simultaneous brain mechanisms underlying the understanding of sounds and human languages. For instance, Teng et al. (2017) showed the simultaneous role of theta and gamma frequency bands during auditory processing, coherently with the “asymmetric sampling in time” (AST) hypothesis proposed by Poeppel (2003). In another study that investigated speech processing, authors revealed that while theta activity was restricted to the auditory cortex, delta band originated in downstream auditory regions, and was modulated by the uncertainty of the stimuli (Donhauser and Baillet 2020).

Altogether, these excellent studies provided a detailed but non-exhaustive review of the brain mechanisms underlying processing of temporal sequences and patterns. In particular, while the review demonstrated how items are chunked and organized in abstract rules through simultaneous mechanisms, it did not conclusively investigate cases where the temporal order of the single items gives rise to a new, superordinate object (sequence) characterized by a novel perception and meaning, which can be encoded and subsequently retrieved or recognized. Musical melodies are ideal examples of such sequences since, depending on the combination of their sounds (items) over time, they are perceived as novel objects conveying meanings that could not be carried by the single sounds alone (Cooke 1959).

Notably, although music neuroscience has rapidly expanded over the last few decades, very little is known on the fast-scale brain mechanisms underlying recognition of musical temporal sequences. Conversely, research in neuroscience of musical memory has provided us with different, highly relevant knowledge. For instance, in an fMRI study, Gaab et al. (2003) asked participants to compare sounds characterized by different pitches. The authors revealed activity especially in superior temporal, supramarginal, and left inferior frontal cortices when participants successfully completed the task. More recently, Kumar et al. (2016) showed the relevant role of primary auditory cortex, hippocampus, and inferior frontal gyrus as well as their connectivity to perform auditory WM-tasks. Similarly, Sikka et al. (2015) asked participants to recognize familiar or unfamiliar music, showing that successful performance was associated to the activation of right superior temporal, bilateral inferior and superior frontal, left middle orbitofrontal, bilateral precentral, and left supramarginal cortices. Besides fMRI, a few studies on musical memory have been conducted using magnetoencephalography (MEG). For instance, Albouy et al. (2013, 2017) investigated the brain activity during memory retention, showing that theta oscillations in the brain dorsal stream of participants predicted their abilities to perform an auditory WM task. In a recent study, Bonetti, Brattico, Carlomagno, et al. (2021a), Bonetti, Bruzzone, Sedghi, et al. (2021b), Bonetti, Brattico, Vuust, et al. (2021c) showed that the first 220 ms of sound encoding presented a large network of connected brain areas such as Heschl’s and superior temporal gyri, frontal operculum, cingulate gyrus, insula, basal ganglia, and hippocampus. Notably, these brain areas were equally central within the network, even if auditory cortex and insula presented stronger activity than the other areas.

In conclusion, previous research has highlighted the brain mechanisms underlying processing of several categories of temporal sequences and musical sounds. However, these studies did not investigate the recognition of previously learned temporal sequences where the order of the single items gave rise to a new, superordinate, global object characterized by novel perception and meaning, arising from musical sequences. In particular, the differential mechanisms associated with processing of the single items of the sequence (local processing) and of memory recognition for the sequence as a whole, novel object (global processing) remain not fully understood. In our study, benefitting from the MEG data constrained by anatomical MRI, we addressed these questions.

## Materials and Methods

### Data availability

The codes are available at the following links:

https://github.com/leonardob92/Brain_Recognition_Temporal_Sequences_Differential_Simultaneous_Processing_Cerebral_Cortex.git

https://github.com/leonardob92/LBPD-1.0.git.

The multimodal neuroimaging data related to the experiment is available upon reasonable request.

### Participants

The study comprised 70 volunteers: 36 males and 34 females (age range: 18–42 years old, mean age: 25.06 ± 4.11 years). All participants were healthy and reported no previous or current alcohol and drug abuse. Moreover, they were not under any kind of medication, declared that they did not have any previous neurological or psychiatric disorder, and reported to have normal hearing. Furthermore, their economic, educational, and social status was homogeneous.

The experimental procedures were carried out complying with the Declaration of Helsinki—Ethical Principles for Medical Research and were approved by the Ethics Committee of the Central Denmark Region (De Videnskabsetiske Komitéer for Region Midtjylland) (Ref 1-10-72-411-17).

### Experimental design and stimuli

**Fig. 1:**
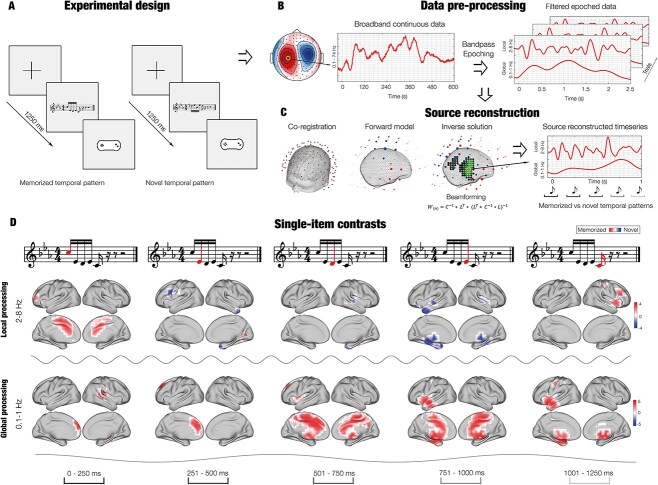
Experimental design, source reconstruction and single-item contrasts. A) After listening to a full musical piece composed by J.S. Bach, participants were presented with a set of melodic excerpts taken from the piece and a series of new melodies. Those excerpts represented temporal sequences built by five items (musical tones) that were labeled by the participants as “previously memorized” (*M*) or “novel” (*N*) using a response pad. B) During the task, participant’s brain activity was recorded with magnetoencephalography. Here we show a graphical depiction of the neural data that was preprocessed, bandpass-filtered in different frequency bands and epoched. This figure depicts analyses for two frequency bands (0.1–1 and 2–8 Hz) since we hypothesized that these two bands were associated to the local and global processing of the temporal sequences. C) Graphical depiction of source reconstruction, computed independently for 0.1–1 and 2–8 Hz. Notably, 0.1–1 Hz indexed the recognition of the whole sequence (global processing), while 2–8 Hz showed the neural responses to each item of the sequence (local processing). D). Contrasts revealed stronger brain activity for *M* versus *N* in 0.1–1 Hz (red), especially for third, fourth and fifth items. Such difference was localized in a large brain network comprising cingulum, inferior temporal cortex, frontal operculum, insula, and hippocampal areas. Conversely, contrasts for 2–8 Hz returned an overall stronger activity for *N* versus *M* (blue), especially in the auditory cortex. The depicted values are t-values obtained by contrasting the brain activity of *M* versus *N*.

To detect the brain signature of temporal sequence recognition, we used an old/new paradigm (Kayser et al. 2003) auditory recognition task during magnetoencephalography (MEG) recording. First, participants listened to four repetitions of a MIDI version of the right-hand part of the whole prelude BWV 847 in C minor composed by J.S. Bach (total duration of about 10 min). Second, they were presented with 80 brief musical excerpts lasting 1,250 ms each and were asked to state whether each excerpt belonged to the prelude by Bach (“memorized” sequence (*M*), old) or it was a novel musical sequence (“novel” sequence (*N*), new) (Fig. 1A). Forty excerpts were taken from the Bach’s piece and 40 were novel. Importantly, the two categories of stimuli (*M* and *N*) were created to be clearly distinguishable in the recognition task (i.e. they were always composed of different musical tones), although they were matched among several variables, to prevent for potential confounds. In fact, *M* and *N* were matched for volume, rhythm, timbre, meter, tempo, tonality, entropy $(H)$, and information content (IC). As mentioned above, the memorized melodies consisted of excerpts of the Bach’s prelude. In this case, we selected one excerpt per musical bar, which corresponded to the first five tones of the bar. Then, we composed the novel musical melodies by using melodic contour and intervals between the notes of the melodies that differed from the memorized musical sequences taken from the Bach’s prelude. The 80 musical sequences are reported in musical notation in Fig. S1.

Regarding IC and $H$, their estimation was done for each tone of the memorized (mean IC: 5.70 ± 1.73, mean $H$: 4.70 ± 0.33) and of the novel musical sequences (mean IC: 5.92 ± 1.81, mean $H$: 4.78 ± 0.35) by using Information Dynamics of Music (IDyOM) (Pearce 2018). This method utilizes machine learning to compute a value of IC for the target note based on a combination of the preceding notes of the musical piece comprising the target note and of a set of rules derived from a large array of prototypical Western musical pieces. Thus, we made sure that the global IC of the musical sequences of our two categories (*M* and *N*) was the same.

On a formal level, the IC corresponds to the minimum number of bits required to encode ${e}_i$ and is described by the following equation (1):

(1) \begin{equation*} \mathrm{IC}\left({e}_i|{e}_{\left(i-n\right)+1}^{i-1}\right)={\log}_2\frac{1}{p\left({e}_i|{e}_{\left(i-n\right)+1}^{i-1}\right)} \end{equation*}

where $p({e}_i|{e}_{(i-n)+1}^{i-1})$ is the probability of the event ${e}_i$ given a previous set of ${e}_{(i-n)+1}^{i-1}$ events.

The entropy provides a measure of the uncertainty/certainty of the upcoming event given the previous set of ${e}_{(i-n)+1}^{i-1}$ events and is computed by the following equation (2):

(2) \begin{equation*} H\left({e}_{\left(i-n\right)+1}^{i-1}\right)=\sum \limits_{e\in A}p\left({e}_i|{e}_{\left(i-n\right)+1}^{i-1}\right) IC\left({e}_i|{e}_{\left(i-n\right)+1}^{i-1}\right) \end{equation*}

According to equation (2), if the probability of a given event ${e}_i$ is 1, the probability of the other events in $A$ will be 0. Thus, $H$ will also be equal to 0 (scenario of maximum certainty). Conversely, if the events are equally probable, $H$ will be maximum (scenario of maximum uncertainty). In conclusion, IDyOM provides an estimation of the predictability of each musical tone, and it has been shown coherent with the human perception (Pearce 2018; Sears et al. 2018).

In the MEG analysis, we used only the correctly recognized trials (mean correct *M*: 78.15 ± 13.56%, mean reaction times (RT): 1,871 ± 209 ms; mean correct *N*: 81.43 ± 14.12%, mean RT: 1,915 ± 135 ms). Both prelude and excerpts were created by using Finale (MakeMusic, Boulder, CO) and presented with Presentation software (Neurobehavioral Systems, Berkeley, CA). After the acquisition of the MEG data, in the same or another day, participants’ brain structural images were acquired by using magnetic resonance imaging (MRI).

### Data acquisition

We acquired anatomical MRI and MEG data in two independent sessions. The MEG data was acquired by employing an Elekta Neuromag TRIUX system (Elekta Neuromag, Helsinki, Finland) equipped with 306 channels. The machine was positioned in a magnetically shielded room at Aarhus University Hospital, Denmark. Data were recorded at a sampling rate of 1,000 Hz with an analogue filtering of 0.1–330 Hz. Prior to the measurements, we set the sound level to 50 dB above the minimum hearing threshold of each participant. Moreover, by utilizing a three-dimensional digitizer (Polhemus Fastrak, Colchester, VT, USA), we registered the participant’s head shape and the position of four headcoils, with respect to three anatomical landmarks (nasion, and left and right preauricular locations).

The location of the headcoils was registered during the entire recording by using a continuous head position identification (cHPI), allowing us to track the exact head location within the MEG scanner at each time-point. We utilized this data to perform an accurate movement correction at a later stage of the data analysis.

The recorded anatomical MRI data corresponded to the structural T1. The acquisition parameters for the scan are reported as follows: voxel size = 1.0 × 1.0 × 1.0 mm (or 1.0 mm^3^); reconstructed matrix size 256 × 256; echo time (TE) of 2.96 ms and repetition time (TR) of 5,000 ms and a bandwidth of 240 Hz/Px. At a later stage of the analysis, each individual T1-weighted MR scan was co-registered to the standard MNI brain template through an affine transformation and then referenced to the MEG sensors space by using the Polhemus head shape data and the three fiducial points measured during the MEG session.

### Data pre-processing

The raw MEG sensor data (204 planar gradiometers and 102 magnetometers) was pre-processed by MaxFilter (Taulu and Simola 2006) for attenuating the interference originated outside the scalp by applying signal space separation. Within the same session, Maxfilter also adjusted the signal for head movement and downsampled it from 1,000 to 250 Hz.

The data were converted into the SPM format and further analyzed in Matlab (MathWorks, Natick, Massachusetts, USA) by using OSL (OHBA Software Library), a freely available toolbox that relies on a combination of FSL (Woolrich et al. 2009), SPM (Penny et al. 2007) and Fieldtrip (Oostenveld et al. 2011), as well as in-house-built functions. A notch filter (48–52 Hz) was applied to correct for possible interference of the electric current. The data was further downsampled to 150 Hz and few segments of the data, altered by large artifacts, were removed after visual inspection. Then, to discard the interference of eyeblinks and heart-beat artifacts from the brain data, independent component analysis (ICA) was used to decompose the original signal into independent components. Then, the components that picked up eyeblink and heart-beat activities were first isolated and then discarded. The signal was rebuilt by using the remaining components (Mantini et al. 2011) and then epoched in 80 trials (one for each musical excerpt) lasting 3,500 ms each (with 100 ms of pre-stimulus time that was used for baseline correction) (Fig. 1B).

### Univariate tests and Monte-Carlo simulations over MEG sensors

Although our primary focus was on the MEG source reconstructed brain data, a first analysis on MEG sensors data was computed, coherently with state-of-the-art recommendation for best practice in MEG analysis (Gross et al. 2013).

Thus, similar to a large number of MEG and electroencephalography (EEG) task-related studies (Gross et al. 2013), we averaged the trials over conditions, obtaining two final mean trials, for *M* and *N*, respectively. Then, we combined each pair of planar gradiometers by root sum square. Afterwards, we performed a *t*-test for each time-point in the time-range 0–2.500 s and each combined planar gradiometer, contrasting *M* versus *N*. To correct for multiple comparisons, we computed Monte-Carlo simulations (MCS) (Kroese et al. 2011) with 1,000 permutations on the clusters of significant results emerged from the t-tests. We considered significant the original clusters that had a size bigger than the 99.9% maximum cluster sizes of the permuted data. Additional details on this widely used procedure can be found in Bonetti et al. (2020), Bonetti, Brattico, Carlomagno, et al. (2021a), Bonetti, Bruzzone, Sedghi, et al. (2021b), Bonetti, Brattico, Vuust, et al. (2021c) and Fernàndez-Rubio, Brattico, et al. (2022a), Fernàndez-Rubio, Carlomagno, et al. (2022b), Fernàndez-Rubio, Olsen, et al. (2022c). This analysis returned a large and robust difference between experimental conditions. Moreover, the brain activity recorded over the MEG channels forming the significant cluster outputted by the MCS analysis outlined a timeseries, which presented two main frequency components. As shown in Fig. S2A, the faster frequency component peaked after the presentation of each of the items forming the sequence, while the slower frequency component accompanied the whole sequence. This evidence was further supported by the computation of complex Morlet wavelet transform (Daubechies 1992) on all MEG sensor data, which highlighted the main contribution of 1 and 4 Hz to the MEG signal recorded during the task (Fig. S2B). In addition, this analysis showed a noticeable yet weaker power around 10 Hz. To be noted, the 10 Hz power was not time-locked to the onset-offset of the musical sequence. Thus, our following analyses primarily focused on two frequency bands defined around 1 and 4 Hz, since they were the frequencies with the strongest power. These two bands were 0.1–1 and 2–8 Hz. Furthermore, we conducted an additional analysis on the frequency range defined around 10 Hz, since it presented a reduced yet distinguishable power. Such band was 8–12 Hz. Importantly, we hypothesized that the frequency bands 2–8 and 0.1–1 Hz indexed the two main processes involved in our experimental task: processing of single items forming the temporal sequence (*i—*local processing) and recognizing the temporal sequence as a comprehensive superordinate object (*ii—*global processing).

### Source reconstruction

**Fig. 2:**
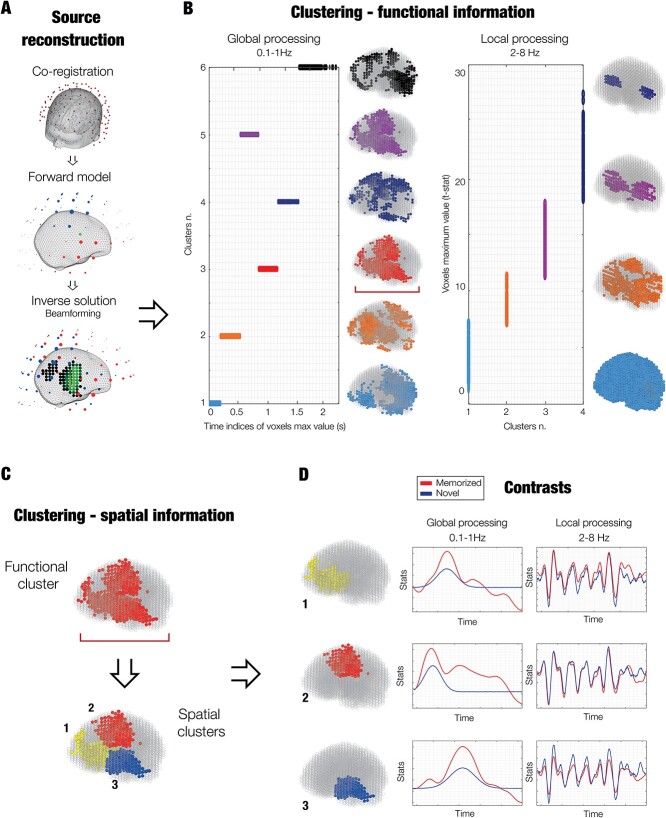
Methods figure showing a graphical depiction of the source reconstruction, *k*-means functional clustering, and contrasts. A) Graphical depiction of the source reconstruction. B) A functional parcellation of the brain based on the activity recorded during the task was estimated. First, *k*-means clustering was computed on functional information of each brain voxel timeseries. Regarding 0.1–1 Hz (indexing the global processing of the sequence), clustering was computed on the time indices of the maximum values of the voxels. Conversely, for 2–8 Hz, indexing the local processing of each object of the sequence), clustering was performed on the maximum values of the voxels. This procedure returns a set of functional parcels. C) A second series of *k*-means clustering was computed on the spatial properties of each of the functional parcels described in (B). Here, for illustrative purposes, we show only one functional parcel (outlined by the red bracket). Such procedure returned a set of new final parcels with the corresponding timeseries considering both functional and spatial information of each of the brain voxels. D) Contrasts between memorized (*M*) and novel (*N*) temporal sequences were computed for each parcel and frequency band.

We used state-of-the-art source reconstruction methods to estimate the sources, which generated the signal that we recorded on the MEG sensors (Fig. 1C and Fig. 2A) (Huang et al. 1999; Hillebrand and Barnes 2005). Importantly, the source reconstruction algorithm has been computed independently for the three frequency bands involved in the study (0.1–1, 2–8 and 8–12 Hz), to characterize the evoked responses to *M* and *N* in these three different frequency bands. Specifically, the following steps were implemented. First, the continuous data (before the epoching) was band-pass filtered into the three frequency bands. Second, the filtered data (independently for the three bands) was epoched. Third, the epoched data were submitted to the source reconstruction algorithm described below.

Such algorithm involves two subsequent steps: (i) designing a forward model and (ii) computing the inverse solution. The forward model is a theoretical model that considers each brain source as an active dipole and describes how the unitary strength of such dipole would be reflected over all MEG sensors (in our case, we utilized both magnetometers and planar gradiometers) (Huang et al. 1999). Here, we employed an 8-mm grid that returned 3,559 dipole locations (voxels) within the whole brain. After co-registering individual structural T1 data with fiducials (information about head landmarks), the forward model was computed by adopting a widely used method called “Single Shell,” presented in details by Nolte (2003). The output of such computation, also referred to as leadfield model, was stored in matrix *L* (sources × MEG channels). In the few cases where structural T1 was not available, we performed the leadfield computation using a template (MNI152-T1 with 8-mm spatial resolution).

The second step of the source reconstruction is to compute the inverse solution (i.e. to estimate the generators of the neural signal on the basis of the brain activity recorded with MEG). In our study, we chose the beamforming, which is one of the most popular and effective algorithms available in the field (Huang et al. 1999; Hillebrand and Barnes 2005). This procedure uses a different set of weights sequentially applied to the source locations for isolating the contribution of each source to the activity recorded by the MEG channels for each time-point (Hillebrand and Barnes 2005; Brookes et al. 2007). On a more technical level, the inverse solution based on beamforming can be described by the following main steps.

First, the data recorded by MEG sensors (*B*) at time *t* can be described by the following equation (3):

(3) \begin{equation*} B_{(t)}=L*Q_{(n_{i},t)}+\xi \end{equation*}

where *L* is the above-described leadfield model, *Q* is the dipole matrix carrying the activity of each active dipole (*q*) over time and *Ɛ* is noise (see Huang et al. (2004) for more details). Thus, to solve the inverse problem, we have to compute *Q*. Using the beamforming, such procedure revolves around the computation of weights that are applied to the MEG sensors at each time-point, as shown for the single dipole *q* in equation (4):

(4) \begin{equation*} q_{(t)}=W^{T}*B_{(t)} \end{equation*}

Indeed, to gain *q*, the weights *W* should be computed (the subscript *T* refers to transpose matrix). To do so, the beamforming relies on the matrix multiplication between *L* and the covariance matrix between MEG sensors (*C*), computed on the concatenated experimental trials. Specifically, for each brain source *n*, the weights *W_n_* are computed as follows:

(5) \begin{equation*} W_{(n)}=(L_{(n)}^T*C^{-1}*L_{(n)})^{-1}*L_{(n)}^{T}*C^{-1} \end{equation*}

To be noted, the computation of the leadfield model was done for the three main orientations of each brain source (dipole), according to Nolte (Nolte 2003). However, before computing the weights, the orientations have been reduced to one by using the singular value decomposition algorithm on the matrix multiplication reported in equation (6). This procedure is widely adopted to simplify the beamforming output (Huang et al. 2004; Woolrich et al. 2011).

(6) \begin{equation*} L=svd(l^{T}*C^{-1}*l)^{-1} \end{equation*}

Here, *l* represents the leadfield model with the three orientations, while *L* the resolved one-orientation model that was used in (5).

Finally, as mentioned above, with regards to the coding implementation of such algorithms, we have used Matlab toolboxes such as OSL, FieldTrip, SPM (functions for MEEG preprocessing and SPM beamforming toolbox) and FSL. Moreover, those codes were complemented by in-house-built scripts and functions.

### Brain activity for each element of the temporal sequence

First, we wanted to detect the brain activity underlying each item of our temporal sequences (Fig. 1D, Figs. S3, S4, Table 1 and Table S1). Here, we computed the absolute value of the reconstructed timeseries since we were interested in the absolute strength of the signal.

To perform first-level analysis for each participant, we employed general linear models (GLMs). Such models were computed on the source reconstructed data for each time-point and brain source (Hunt et al. 2012). The GLMs returned the main effect (contrasts of parameters estimate (COPEs)) of *M* and *N* as well as their contrast. They were employed since they allowed to obtain main effects that were crucially adjusted by the variance across participants. These results were submitted to a second-level analysis, employing one-sample t-tests with spatially smoothed variance obtained with a Gaussian kernel (full-width at half-maximum: 50 mm) (Huang et al. 2004).

Here, we were interested in observing the different brain activity underlying recognition of *M* versus *N* temporal sequence, independently for each frequency band and item (musical tone) forming the sequence. Thus, we computed 15 (5 tones × 3 frequency bands) cluster-based Monte-Carlo simulations (MCS) on the second-level (group-level) analysis results averaged over the five time-windows corresponding to the duration of the musical tones. The MCS analysis comprises 1,000 permutations and a cluster forming threshold of *P* < 0.05 (from the second-level *t*-tests). Specifically, the MCS test consisted of detecting the spatial clusters of significant values in the original data. Then, such data were permuted, and the spatial clusters of the permuted significant values were detected. This procedure was computed several times (e.g. 1,000) and gave rise to a reference distribution of cluster sizes detected for each permutation. Finally, the original cluster sizes were compared to the reference distribution. The original clusters were considered significant if the cluster sizes of the permuted data were bigger than the original cluster sizes less times than the MCS α level. In this case, since we computed the analysis 15 times, we corrected for multiple comparisons by dividing the standard MCS α level (=0.05) by 15, resulting in an updated MCS α = 0.003 (i.e. original clusters were significant if their sizes were larger than the 99.7% of the permuted cluster sizes).

Since one of the frequency ranges used in this study was rather low (0.1–1 Hz), we recomputed the source reconstruction and the contrasts between *M* and *N* for 0.1–1 Hz using three different baselines (500, 1,000, and 2,000 ms). This was done to demonstrate that our original results were not driven by the length of the baseline. The results of this procedure are depicted in Fig. S5 and reported in detail in Table S2.

### *K*-means functional clustering

To complement our previous results and provide a more detailed description of the spatial extent of the active brain sources as well as their activity over time, we defined a functionally based parcellation of the brain. We adopted a so-called *k*-means functional clustering, consisting of a series of *k*-means clustering algorithms. Sinaga and Yang (2020) performed on functional and spatial information of each of the reconstructed brain sources (voxels) timeseries. This approach has been followed for the two frequency bands that returned the strongest results in our previous analyses and that were associated either to the single items of the sequence or to the whole sequence, namely 0.1–1 and 2–8 Hz.

Specifically, as a first step the *k*-means functional clustering algorithm computed a clustering on basic functional parameters such as peak values and the corresponding indices of the voxels timeseries. We refer to this step as functional clustering. This procedure returned a set of independent parcels grouped according to the functional profiles of the brain voxels. Indeed, such parcels could either contain voxels that peaked approximately at the same time (Fig. 2B, left) or with similar absolute strength (Fig. 2B, right). As conceivable, clustering on the maximum timeseries indices is suggested when the brain activity is localized in different regions at different times. Conversely, when the activity is highly correlated over most of the brain voxels, clustering should be done on maximum timeseries values and would help to identify the core generators of the neural signal. In this study, 0.1–1 Hz (global processing of the sequence) presented different peaks of activity shifted over time and thus was clustered considering the time-indices of such peaks. Differently, 2–8 Hz (local processing of the sequence) showed very correlated activity and was therefore clustered using the absolute values of such peak activity. As widely done in clustering analysis (Garcia-Dias et al. 2019), also in our case it was beneficial to compute the clustering algorithm on a sequential set of *k* clusters (from *k* = 2 to 20). Then, the best clustering solution was decided on the basis of well-known evaluation strategies (heuristics) such as the elbow method/rule (Liu and Deng 2021) and the silhouette coefficient (Al-Zoubi and Al Rawi 2008). The elbow method consists in plotting the sum of squared errors (SSE) of the elements belonging to the clusters with respect to the cluster’s centroids, as a function of the progressively more numerous cluster solutions. Then, the method suggests to visually identify the “elbow” of the curve as the number of clusters to use. The silhouette coefficient is a value (ranging from −1 to +1) showing the similarity of an element with its own cluster (cohesion) when compared to other clusters (separation). A high silhouette coefficient value indicates that the element is well matched to its own cluster and poorly to the neighboring clusters.

Once the best functional clustering solution is decided, a second clustering with regards to spatial information should be computed (spatial clustering, Fig. 2C). Indeed, brain activity is mainly described by two parameters, spatial locations, and variation over time. Clustering the original brain voxels into distinct functional parcels may return large parcels involving a network of spatially separated brain areas that are e.g. active at the same time. Thus, to define a better parcellation, it is beneficial to conduct clustering analysis also on the spatial coordinates of each of the functional parcels. In our study, we considered the three-dimensional spatial coordinates (in MNI space) of the voxels forming each of the functional parcels. This clustering computation was performed for a sequential set of *k* clusters solutions (from *k* = 2 to 10), for one parcel at a time. As for the functional clustering, we evaluated the best solution for the spatial clustering by using the elbow rule and the silhouette coefficient. The *k*-means functional clustering was complete once this procedure was performed on all functional parcels, suggesting an effective parcellation for the experimental task based on both functional and spatial information (examples are reported in Fig. S7, Tables S3 and S4 for 0.1–1 Hz and Fig. S8, Tables S5, S6 and S7 for 2–8 Hz). As a last step, the timeseries of the brain voxels belonging to each parcel were averaged together to provide a final timeseries for the parcel. Additional information on the *k*-means functional clustering is reported in the Supplementary Materials.

### Contrasts over time for each parcel

**Fig. 3:**
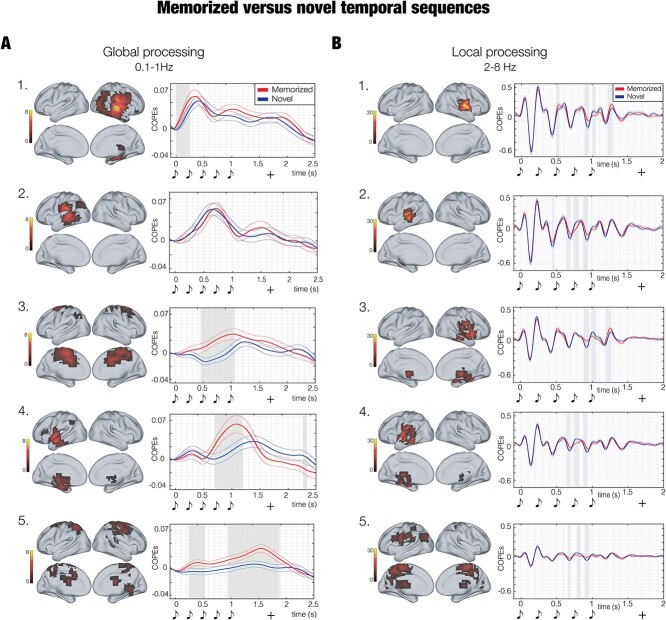
Contrasts between *M* versus *N* temporal sequences for the main functionally derived ROIs. A) Timeseries and contrasts between *M* (red line) versus *N* (blue line) temporal sequences for the main functional brain parcels (global processing—0.1–1 Hz). The plots show a stronger activity for *M* over *N* in a network of sequentially recruited ROIs (B). Timeseries and contrasts between *M* (red line) versus *N* (blue line) temporal sequences for the main functional brain parcels (local processing—2-8 Hz). The plots illustrate a stronger activity for *N* over *M* in nearly all ROIs. The graphical depiction of musical tones indicates the onset of the objects forming the temporal pattern, while the “+” shows the mean reaction time of participants’ response. The colourbars refer to the *t*-values obtained from second-level analyses. The gray shadows indicate the time-windows of significant differences between *M* and *N* timeseries.

Here, the *k*-means functional clustering was performed on the group-level main effects of *M* and *N* averaged together. Then, to obtain the main effect of *M* and *N* for each parcel and participant, we averaged the first-level main effect of *M* and *N* (from the GLMs) over the brain voxels belonging to each of the functional parcels. This resulted in a new timeseries for each participant, functional parcel, and experimental condition (*M* and *N*). Such timeseries were submitted to univariate contrasts (*M* versus *N*; Fig. 2D, methods, and Figs. S9 and S10, results). Specifically, for each parcel and time-point, we computed one two-sample *t*-test (threshold *P* < 0.05) contrasting the main effect of *M* versus *N*. Then, we corrected for multiple comparison by using a two-dimensional MCS approach with 1,000 permutations (MCS *P* < 0.001). More details on this widely adopted statistical procedure can be found in Bonetti et al. (2020), Bonetti, Brattico, Carlomagno, et al. (2021a), Bonetti, Bruzzone, Sedghi, et al. (2021b), Bonetti, Brattico, Vuust, et al. (2021c). As done for the other analyses, such operation was observed for the two main frequency bands investigated in the study (Fig. 3 and Table S8).

## Results

### Experimental design and MEG sensors analysis

In the first place, after preprocessing the MEG data (see Fig. 1A and B and Materials and Methods for details), we contrasted the brain activity underlying recognition of *M* versus *N*, which was recorded by the MEG sensors. This procedure returned a large significant cluster (*P* < 0.001, cluster size *k* = 2,117, mean *t-*value = 3.29, time = 0.547–1.180 s), showing stronger brain activity for *M* versus *N*. Moreover, the brain activity recorded over the MEG channels forming such significant cluster outlined a timeseries, which presented two main frequency components. As shown in Fig. S2A, the faster frequency component peaked after the presentation of each of the items forming the sequence, while the slower frequency component accompanied the whole sequence. This evidence was further supported by the computation of complex Morlet wavelet transform on the evoked responses recorded by all MEG sensor data, which highlighted the main contribution of 1 and 4 Hz to the MEG signal recorded during the task (Fig. S2B). Thus, our following analyses primarily focused on two frequency bands defined around 1 and 4 Hz, which were 0.1–1 and 2–8 Hz. Importantly, we hypothesized that the frequency bands 2–8 and 0.1–1 Hz indexed the two main processes involved in our experimental task: processing of single items forming the temporal sequence (i*—*local processing) and recognizing the temporal sequence as a comprehensive superordinate object (ii*—*global processing).

### Source reconstructed brain activity and single-item analysis

We contrasted the reconstructed brain activity underlying *M* versus *N* sequences (see Materials and Methods for details). Different results emerged for the main two frequency bands (0.1–1 and 2–8 Hz). Brain activity for 0.1–1 Hz was stronger for *M* versus *N*, especially during processing of the last three items of the sequence. As depicted in Fig. 1D, Figs S3–S5, such activity delineated a widespread brain network underlying the global processing of the sequence, involving brain regions related to memory and evaluative processes such as cingulate gyrus, hippocampus, insula, frontal operculum, and inferior temporal cortex (MCS *P* < 0.001). Conversely, brain activity for the 2–8 Hz band was overall stronger for *N* versus *M* and mainly involved auditory cortices (MCS *P* < 0.001). Statistics of the peak significant brain voxels for frequencies 0.1–1 and 2–8 Hz are reported in Table 1, while extensive results for the three frequency bands are described in Tables S1 and S2.

**Table 1:** Peak brain activity underlying recognition of each item (musical tone) of the temporal sequences

**0.1–1 Hz**						**2–8 Hz**					
**Brain area**	**Hemisphere**	**t**	**MNI coordinates**			**Brain area**	**Hemisphere**	**t**	**MNI coordinates**		
			***x***	***y***	***z***				***x***	***y***	***z***
**Tone 1**											
Rolandic Ope	R	4.44	42	−30	16	Cing Mid	R	4.48	2	2	40
Heschl	R	4.26	42	−30	8	Cing Mid	R	4.23	2	10	40
Temporal Sup	R	4.16	50	−30	16	Cing Mid	L	4.17	−6	2	40
Temporal Sup	R	4.04	42	−38	16	Cing Mid	R	4.16	2	10	32
**Tone 2**											
Frontal Sup	L	4.00	−14	34	40	Tempr Pol Sup	R	−3.88	34	10	−32
Frontal Sup	L	3.98	−14	34	32	Tempr Pol Sup	R	−3.46	26	10	−32
Frontal Sup	L	3.92	−14	26	40	Front Inf Ope	L	−3.45	−38	2	24
Frontal Sup	L	3.78	−14	42	40	Tempr Pol Mid	R	−3.37	42	10	−32
**Tone 3**											
Precuneus	R	3.89	2	−46	48	Temporal Sup	R	−3.30	50	−22	8
Cing Mid	R	3.80	2	−38	48	Temporal Sup	R	−3.19	58	−22	8
Cing Mid	R	3.62	2	−22	48	Temporal Sup	R	−2.78	50	−22	0
Cing Mid	R	3.60	2	−30	48	Heschl	R	−2.67	42	−22	8
**Tone 4**											
Temporal Mid	L	5.05	−46	−6	−16	ParaHippocamp	L	−3.89	−22	−30	−16
Insula	L	4.93	−38	−6	−8	ParaHippocamp	L	−3.86	−30	−30	−16
Temporal Mid	L	4.81	−46	−14	−16	Tempr Pol Mid	L	−3.74	−46	10	−32
Cing Mid	R	4.76	2	−6	40	Tempr Pol Mid	L	−3.70	−38	10	−32
**Tone 5**											
Insula	L	5.48	−38	2	−8	Front Inf Tri	R	3.41	42	26	24
Putamen	L	5.27	−30	2	−8	Putamen	R	3.36	34	2	0
Temporal Mid	L	5.26	−46	−6	−16	Insula	R	3.28	42	10	0
Temp Pol Mid	L	5.24	−46	2	−16	Postcentral Gyr	R	3.23	34	−30	48

Brain areas refer to the automatic anatomic labelling (AAL) parcellation labels, while *t* indicates the *t*-value obtained by contrasting known versus unknown temporal sequences.

### Contrasts on functionally derived ROIs

To fully characterize the spatiotemporal unfolding of brain activity over time, we defined a functionally based parcellation of the brain using *k*-means functional clustering (see Materials and Methods and Fig. 2 and Fig. S6 for details), independently for 0.1–1 and 2–8 Hz frequency bands. This resulted in a new timeseries for each participant, functional parcel, and experimental condition (*M* and *N*), which were submitted to univariate contrasts (*M* versus *N*) (Fig. 3) and corrected for multiple comparisons using cluster-based MCS.

Similar to our previous analysis, the strongest brain activity in the slower band was detected for *M*. Remarkably, expanding on our first analysis, these new results highlighted a series of sequentially active brain parcels accompanying the processing of the temporal sequence. As shown in Fig. 3A, the brain presented an initial activity in the right auditory cortex characterized by a slightly stronger power for *M* versus *N* (Fig. 3A, parcel 1: *P* < 0.001, cluster size *k* = 39; mean *t-*value = 2.72; time from first object onset: 0–0.25 s). Next, we observed neural activity in the left auditory cortex but no significant differences between experimental conditions (Fig. 3A, parcel 2). Starting between the second and third items and peaking during the fifth item of the temporal sequence, we observed a burst of activity in the cingulate gyrus, which was stronger for *M* versus *N* (Fig. 3A, parcel 3: *P* < 0.001, *k* = 92; *t-*value = 2.73; time: 0.45–1.05 s). With a slight delay, a similar profile emerged for a larger brain parcel comprising insula, the anterior part of the inferior temporal cortex, hippocampus, and frontal operculum. Once again, *M* was largely stronger than *N* (Fig. 3A, parcel 4: *P* < 0.001, *k* = 77; *t-*value = 2.79; time: 0.69–1.19 s). Finally, peaking just before the mean reaction time for participants’ categorization of the pattern, a stronger activity in post-central gyrus and sensorimotor cortex was observed for *M* versus *N* (Fig. 3A, parcel 5, main cluster: *P* < 0.001, *k* = 142; *t-*value = 2.68; time: 0.94–1.88 s).

Conversely, the analysis for 2–8 Hz band showed several significant clusters of stronger activity for *N* versus *M* around the sharp peaks of the timeseries. Notably, compared to our first analysis for the 5 items of the temporal sequence, this second procedure clearly outlined the temporal extent of such difference, which corresponded to the last three tones of the temporal sequences. Specifically, such differences involved right (Fig. 3B, parcel 1, main cluster I: *P* < 0.001, *k* = 11, *t-*value = 3.51; time: 0.89–0.95 s; II: *P* < 0.001, *k* = 11; *t-*value = 2.22; time: 1.21–1.28 s) and left primary auditory cortices (Fig. 3B, parcel 2, main cluster I: *P* < 0.001, *k* = 12, *t-*value = −3.70; time: 0.74–0.81 s; II: *P* < 0.001, *k* = 12; *t-*value = 3.09; time: 0.87–0.95 s; III: *P* < 0.001, *k* = 9; *t-*value = 2.90; time: 0.64–0.69 s). With a reduced strength, similar clusters of activity have been observed for right (Fig. 3B, parcel 3, main cluster I: *P* < 0.001, *k* = 13, *t-*value = 3.08; time: 1.19–1.27 s; II: *P* < 0.001, *k* = 12; *t-*value = 3.61; time: 0.89–0.96 s) and left secondary auditory cortex and hippocampal areas (Fig. 3B, parcel 4, main cluster I: *P* < 0.001, *k* = 12, *t-*value = −2.97; time: 0.74–0.81 s; II: *P* < 0.001, *k* = 10; *t-*value = 2.86; time: 0.87–0.93 s) and cingulate (Fig. 3B, parcel 5, main cluster I: *P* < 0.001, *k* = 10, *t-*value = 3.03; time: 0.90–0.96 s; II: *P* < 0.001, *k* = 9; *t-*value = −2.29; time: 0.79–0.84 s). Additional details on these contrasts are reported in Tables S8, and extensively depicted in Figs. S9 and S10.

**Fig. 4:**
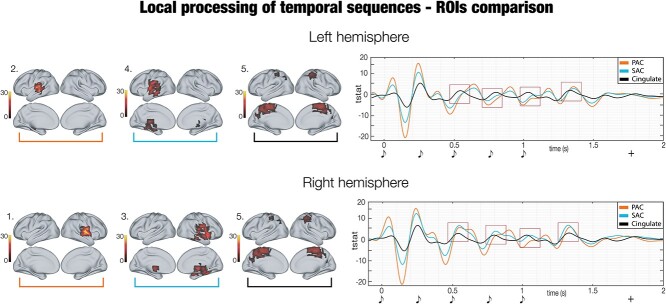
Focus on local processing (main ROIs). Deepening on three main parcels (primary auditory cortex (PAC), secondary auditory cortex and hippocampal areas (SAC) and cingulate) of the local processing. The image highlights the different behavior of PAC versus SAC and Cingulate, especially in the right hemisphere. It shows that higher-order areas (SAC and Cingulate) are more implicated than lower order ones (PAC) in the generation of the P300 component in response to each sound of the pattern (as outlined by the red squares). The graphical depiction of musical tones indicates the onset of the objects forming the temporal pattern, while the “+” shows the mean reaction time of participants’ response. Colourbars refer to the *t*-values obtained from second-level analyses.

In conclusion, Fig. 4 qualitatively illustrates an interesting phenomenon. While the “wavelet” response to the first sound showed very similar activity over primary (parcel i) and secondary auditory cortices, insula, hippocampal areas (parcel ii), and cingulate (parcel iii), the peaks for the following sounds showed a different trend, especially in response to the third and fourth items of the sequence. In this case, secondary auditory cortices, insula, hippocampal areas, and cingulate seemed to peak before the primary auditory cortex. However, contrary to what it may appear initially, this may not indicate a faster response of those areas. Indeed, looking, for example, at the peaks around 0.5 s (first red square in Fig. 4), the first peak (mainly occurring for secondary auditory cortices, insula, hippocampal areas and cingulate) should correspond to the P300 component to the second sound of the pattern, while the second peak (mainly occurring for primary auditory cortex) may be the P50 to the third sound. An analogous phenomenon happened for the following items of the sequence (as outlined by the other red squares). This may suggest that while the contribution of the primary auditory cortex was stronger for the first components (i.e. P50 and N100), which indexed lower-level processes, later components such as P300 may be mainly generated by higher order areas such as secondary auditory cortices, insula, hippocampal areas, and cingulate cortices. In the current state, this is only a qualitative observation that calls for future studies aiming to specifically and quantitively investigate this phenomenon.

## Discussion

This MEG study using musical sequences revealed the dual simultaneous processing in the brain associated with the recognition of auditory temporal sequences. On the one hand, the presentation of local, single items forming the sequence was linked to a rapid, oscillatory, local processing driven by sensorial cortices. This processing was stronger for the recognition of the sounds forming the novel versus memorized musical sequences. On the other hand, the processing of the global, whole temporal sequence was associated with a simultaneous global, slow processing involving a widespread network of sequentially active high-order brain areas. In this case, the brain activity was largely stronger for memorized versus novel sequences.

This dual simultaneous processing was particularly evident in correspondence to the presentation of the last three tones of the sequence, suggesting that at least two or three tones are required by the brain to start the recognition process. Here, the brain recruited a widespread network of areas largely related to memory, attention, audition, and decision-making. Such brain areas were hippocampus (Knierim 2015), cingulate gyrus (Rolls 2019; Pando-Naude et al. 2021; Criscuolo et al. 2022), inferior temporal cortex (Conway 2018), frontal operculum (Indefrey et al. 2001; Behroozmand et al. 2015), insula (Uddin 2015), and primary and secondary auditory cortex (Elhilali et al. 2004). Notably, both processes (global and local) involved approximately the same brain regions but depended on different frequencies of the neural evoked responses. Furthermore, the local processing relied mainly on sensorial cortices (e.g. auditory cortex), while the global processing presented a wider recruitment of higher order brain areas such as cingulate, inferior temporal cortex and hippocampus.

Strikingly, auditory temporal sequence recognition was associated with a cascade of progressively slower events rewiring a chain of low- to high-order brain regions. This evidence, observed for the 0.1–1 Hz band, may indicate that the brain tracks and progressively constructs a meaningful understanding of the unfolding temporal sequence by recruiting a hierarchical pathway of subsequently active regions. Conversely, activity in the 2–8 Hz band showed a complementary profile, which peaked slightly after each item of the temporal sequence. Such evidence suggests that, while the 0.1–1 Hz band may be implicated in achieving a comprehensive understanding of the whole sequence (global processing), the 2–8 Hz band may elaborate independently on the single items (local processing). However, our results cannot conclusively tell whether such bands represented internal brain mechanisms or were simply stimulus driven. Future research will be needed to address this important question.

The main novelty of our results relates to the differential strength of the brain signal observed for the two frequency bands in relation to our experimental conditions (*M* and *N*). Indeed, while the 0.1–1 Hz band presented a stronger power for the memorized sequences, the 2–8 Hz band showed greater responses for the novel ones. This finding may be seen in light of the predictive coding theory (Friston 2012; Vuust et al. 2012; Koelsch et al. 2019), which posits that the brain is constantly updating internal models to predict environmental information. Here, when the brain is recognizing the temporal sequences (e.g. around tones number two and three of our sequences), it might formulate better predictions of the upcoming, previously memorized, items completing the sequences. Thus, such items would require a lower local processing, as we observed experimentally. Interestingly, although mainly localized in primary auditory cortex, the neural sources of 2–8 Hz band activity were also placed in hippocampal areas, secondary auditory cortex, insula, and cingulate. As previously mentioned, this evidence suggests that roughly the same brain regions generated two simultaneous frequency bands characterized by a very different functional profile, indexing the local and global processing of the temporal sequence. On top of this, with regards to local processing, our results show that the elaboration of each sound gave rise to a wavelet-like timeseries with three main peaks (components). Here, the lower level elaboration of the sounds indexed by the first components (i.e. P50 and N100 (Coles and Rugg 2008)) originated mainly in the primary auditory cortex. Conversely, later components such as P300 (Coles and Rugg 2008) were generated especially by higher order areas such as secondary auditory cortices, insula, hippocampal regions, and cingulate. Remarkably, such phenomenon became more evident following the unfolding of the temporal sequence, suggesting that a progressively more refined elaboration of the single items may be essential for the brain to comprehend the meaning of the whole temporal sequence.

On another note, several previous studies described global and local processing in terms of different locations of the neural signal (i.e. primary sensorial cortices preceded higher-order brain areas in the elaboration of incoming stimuli (Qiu and Von Der Heydt 2005). Conversely, in our study we showed that the same brain regions operated these 2 processes (global and local) at the same time, using two concurrent frequency bands, perhaps suggestive of complex multiplexing. These results are coherent with previous research, which showed concurrent brain processes of the same items in auditory perception and language comprehension (Giraud and Poeppel 2012). In addition, some of the previous studies that investigated auditory processing and memory for sound information reported slow evoked responses similar to our slow, global processing. For instance, Picton (1978) provided evidence of auditory sustained electric potential in the human brain in response to sounds. More recently, Bidet-Caulet et al. (2007), using intracranial EEG, showed that selective attention for sound information was associated with sustained evoked responses in secondary auditory brain regions. Similarly, Grimault et al. (2014), studying auditory short-term memory, reported sustained brain activity in frontal, temporal and parietal regions when several auditory items were held in memory. In line with these findings, Albouy and colleagues showed slow evoked responses when participants were asked to perform auditory working memory tasks. They reported the slow, sustained brain responses especially in relation to retention and manipulation of auditory stimuli (Albouy et al. 2013, 2017).

Finally, our findings related and expanded concepts of the well-known two-stream hypothesis of the brain (Goodale and Milner 1992; Whitwell et al. 2014). Such conceptualization proposed 2 main pathways for high-order elaboration of visual and auditory information. On the one hand, the ventral stream leads from sensorial areas (e.g. visual and auditory cortices) to the medial temporal lobe, processing features mainly associated to object recognition (Goodale and Milner 1992; Weiller et al. 2021). On the other hand, the dorsal stream brings information from sensory cortices to the parietal lobe, elaborating spatial features of the stimuli (Arbib 2017). Coherent with this hypothesis, our results highlighted several brain regions of the ventral stream that are implicated in recognition processes, such as hippocampal areas, frontal operculum, and inferior temporal cortex. Remarkably, however, our results further expanded previous knowledge on the two-stream hypothesis by providing at least three crucial, conclusive remarks. (i) The brain recognition of temporal sequences presented unique spatial–temporal features, which were not shared with the identification of single items or synchronous patterns. (ii) In addition to the brain regions involved in the two-stream hypothesis, our findings showed the privileged role of cingulate gyrus to achieve auditory temporal sequence recognition. (iii) Finally, the recognition of sequences unfolding over time involved a dual simultaneous processing of the same items, which the brain interpreted concurrently as individual pieces of information (local processing) and elementary parts of a larger whole (global processing). Notably, our study showed that the local processing was highly relevant for the novel auditory information, while the previously memorized musical sequences were recognized through a strong involvement of the brain global processing.

Future research is called to further investigate this topic by studying the brain mechanisms underlying recognition of non-musical temporal sequences (e.g. sequences of numbers, words, and visual elements). In addition, based on the well-known differences in cognitive abilities among diverse categories of people (e.g. older versus younger adults (Fernàndez-Rubio, Brattico, et al. 2022a; Fernàndez-Rubio, Olsen, et al. 2022c), musicians versus non-musicians (Criscuolo et al. 2019; Bonetti, Brattico, Vuust, et al. 2021c), and healthy individuals versus patients (Valenzuela and Sachdev 2006)), future studies should explore the impact of age and clinical conditions on the brain mechanisms underlying temporal sequences recognition.

## Author contributions

Conceptualization: LB, EB, MLK, PV; Methodology: LB, MLK, DP, GDE; Software: LB; Analysis: LB; Investigation: LB, GDO; Resources: MLK, PV, EB, LB; Data curation: LB; Writing—Original draft: LB; Writing—Review and editing: LB, SEPB, EB, DP, GDE, GDO, PV; Visualization: LB, SEPB; Supervision: MLK, PV, DP, EB; Project administration: LB, MLK, PV, EB; Funding acquisition: LB, PV, MLK.

## Supplementary Material

TableS1_bhac439

TableS2_bhac439

TableS3_bhac439

TableS4_bhac439

TableS5_bhac439

TableS6_bhac439

TableS7_bhac439

TableS8_bhac439

TableS9_bhac439

FigureS1_bhac439

FigureS2_bhac439

FigureS3_bhac439

FigureS4_bhac439

FigureS5_bhac439

FigureS6_bhac439

FigureS7_bhac439

FigureS8_bhac439

FigureS9_bhac439

FigureS10_bhac439

FigureS11_bhac439

FigureS12_bhac439

SupplementaryMaterial_bhac439
