# Supplementary figures and images for: Brain recognition of previously learned versus novel temporal sequences: a differential simultaneous processing
Source: Cereb Cortex. 2022 Nov 8;33(9):5524–37. doi: 10.1093/cercor/bhac439 (PMC10152090; doi:10.1093/cercor/bhac439)

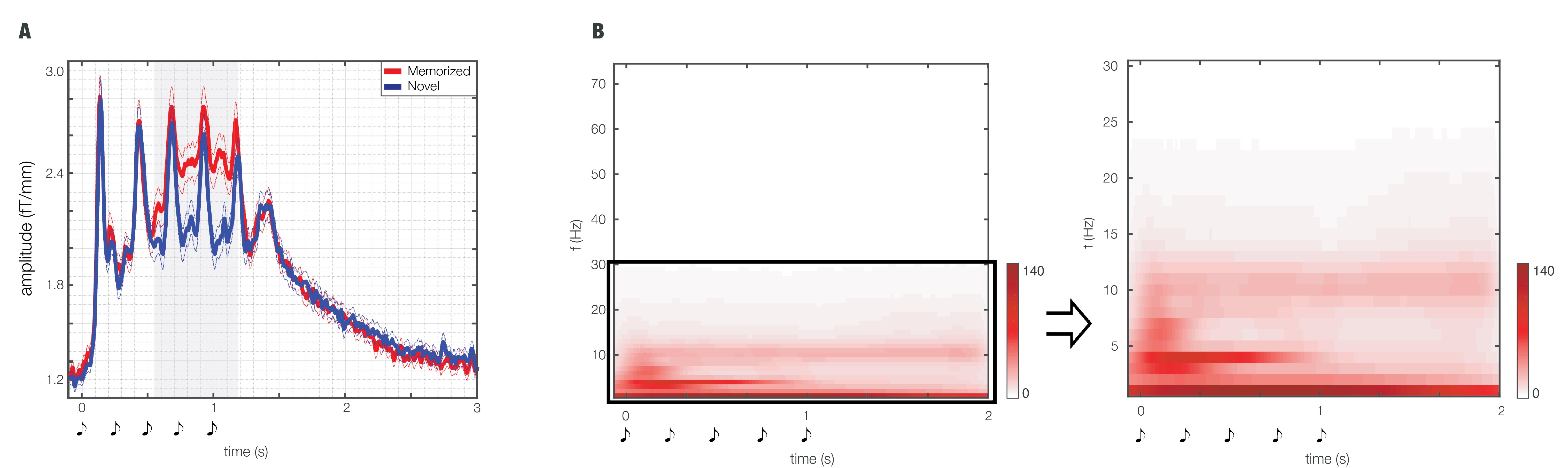

Supplement: FigureS2_bhac439 [file figures2_bhac439.jpeg]

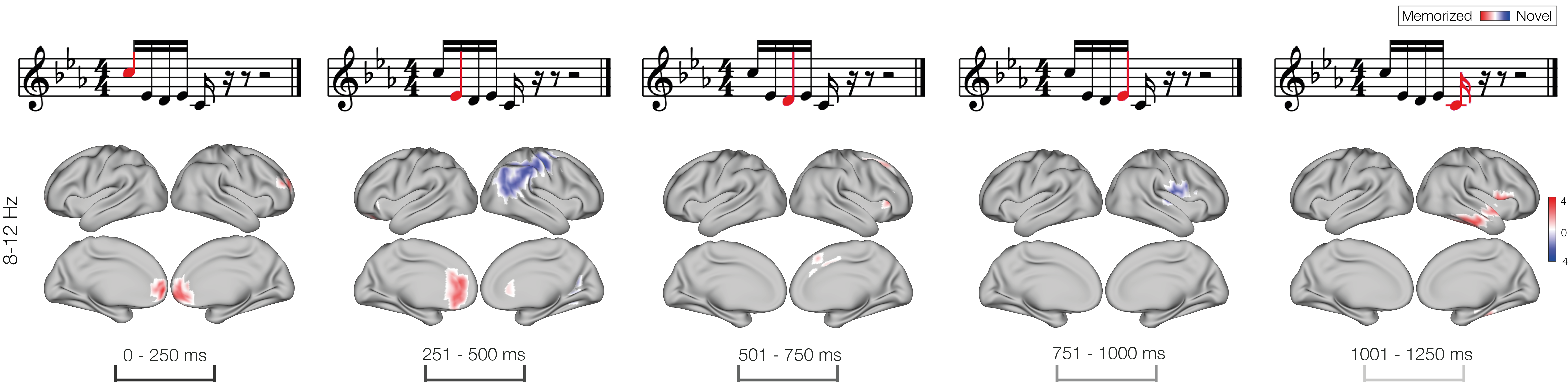

Supplement: FigureS4_bhac439 [file figures4_bhac439.zip › FigureS4_bhac439.tiff]

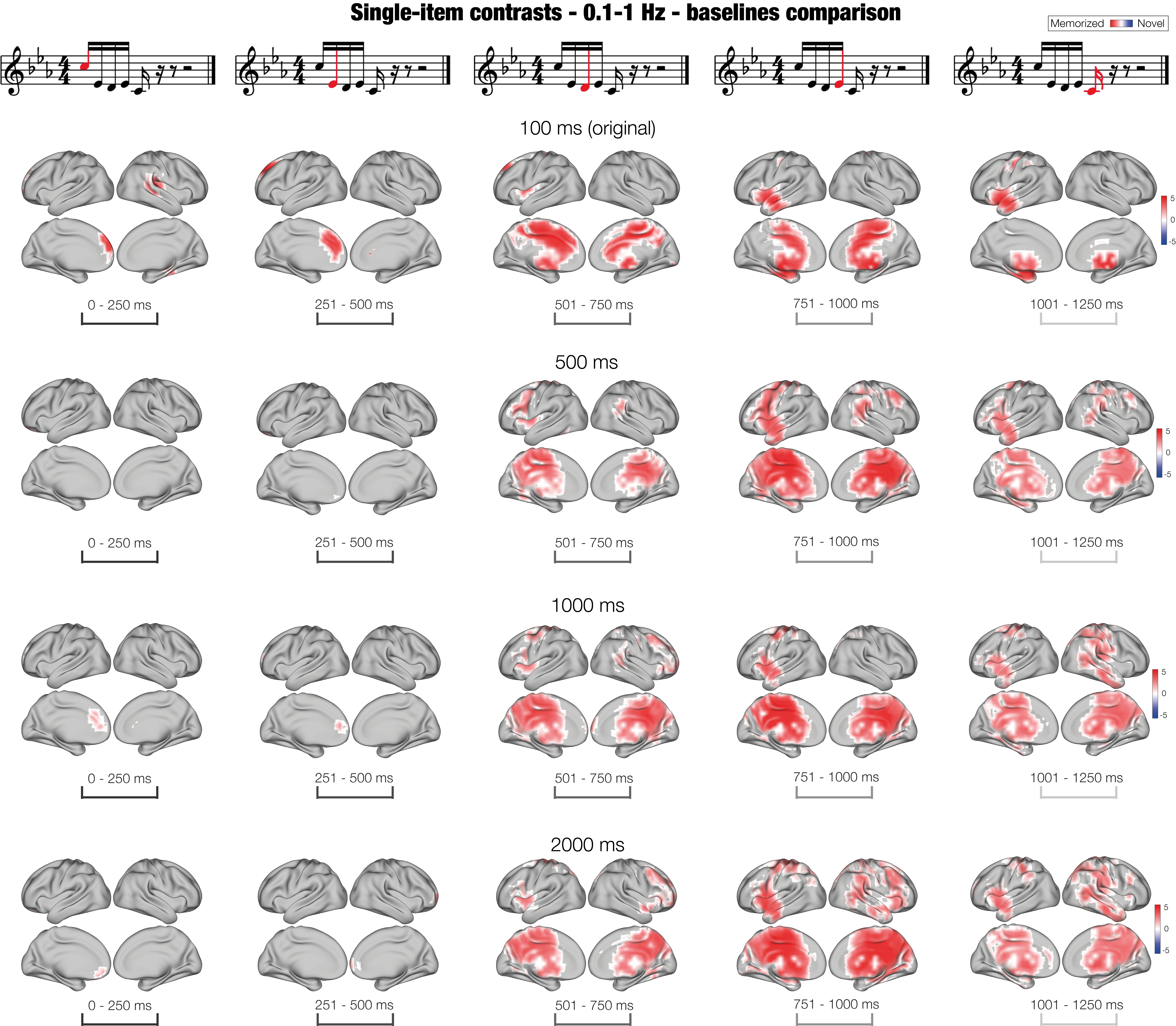

Supplement: FigureS5_bhac439 [file figures5_bhac439.zip › FigureS5_bhac439.tiff]

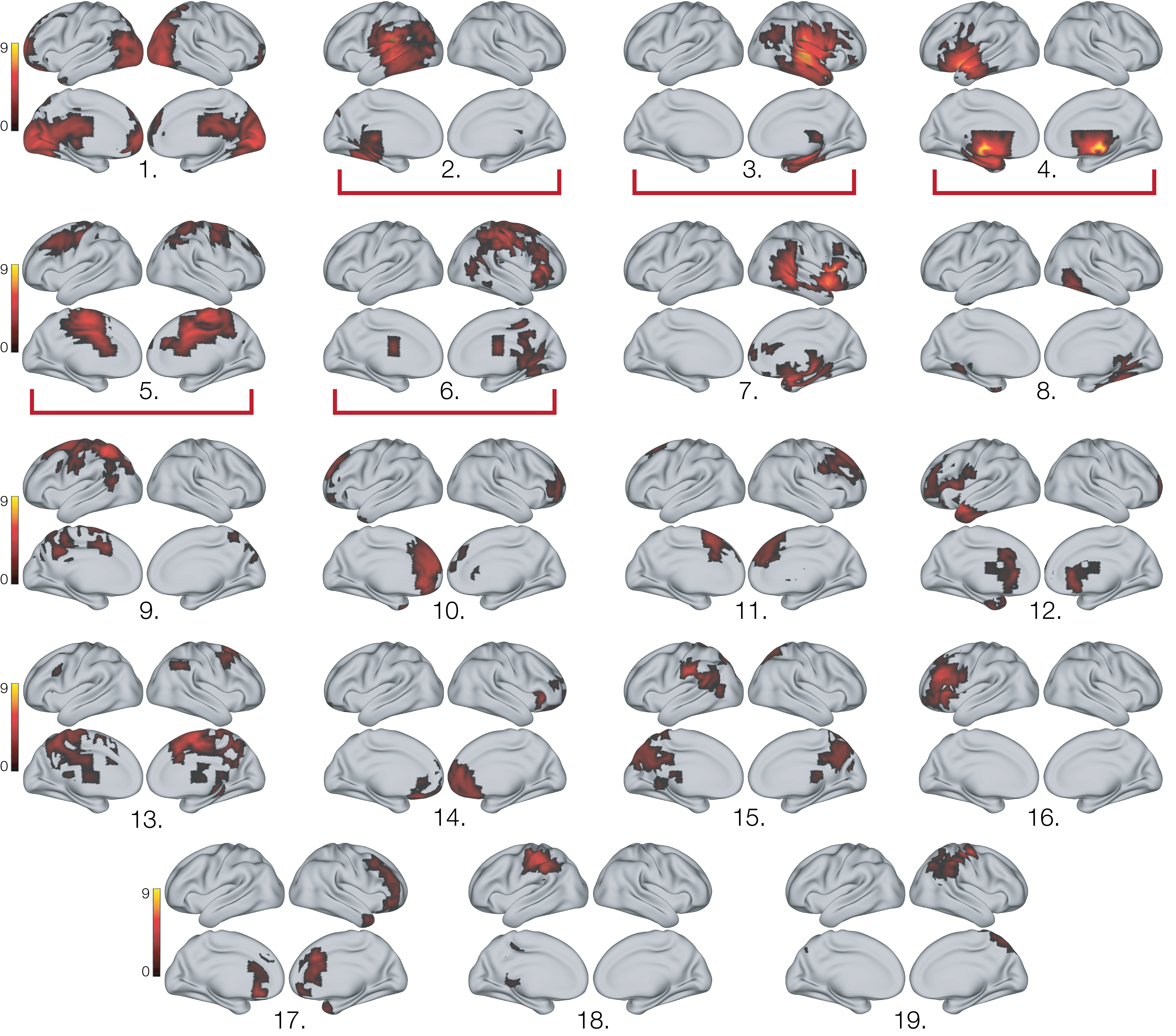

Supplement: FigureS7_bhac439 [file figures7_bhac439.zip › FigureS7_bhac439.tiff]

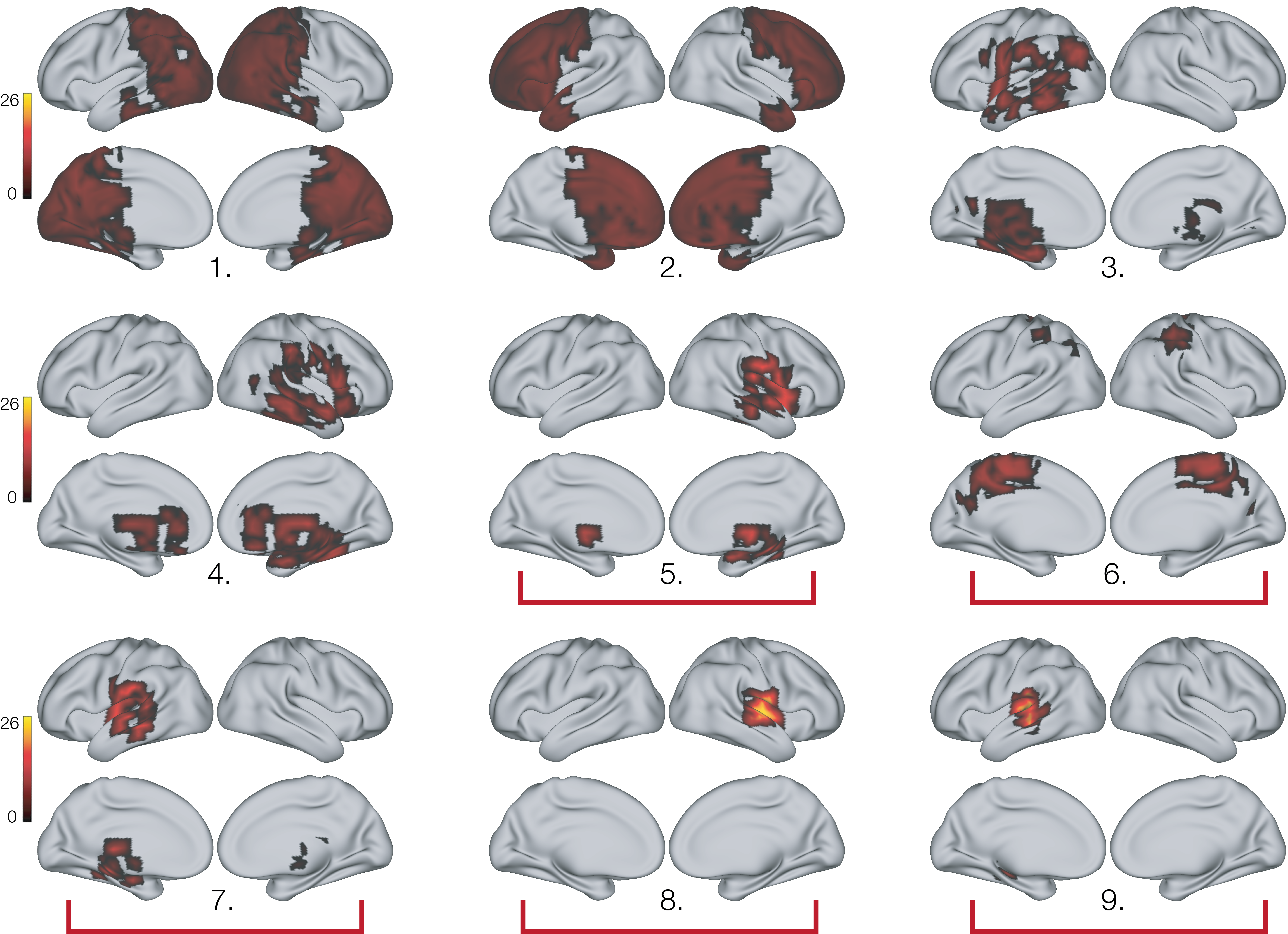

Supplement: FigureS8_bhac439 [file figures8_bhac439.zip › FigureS8_bhac439.tiff]

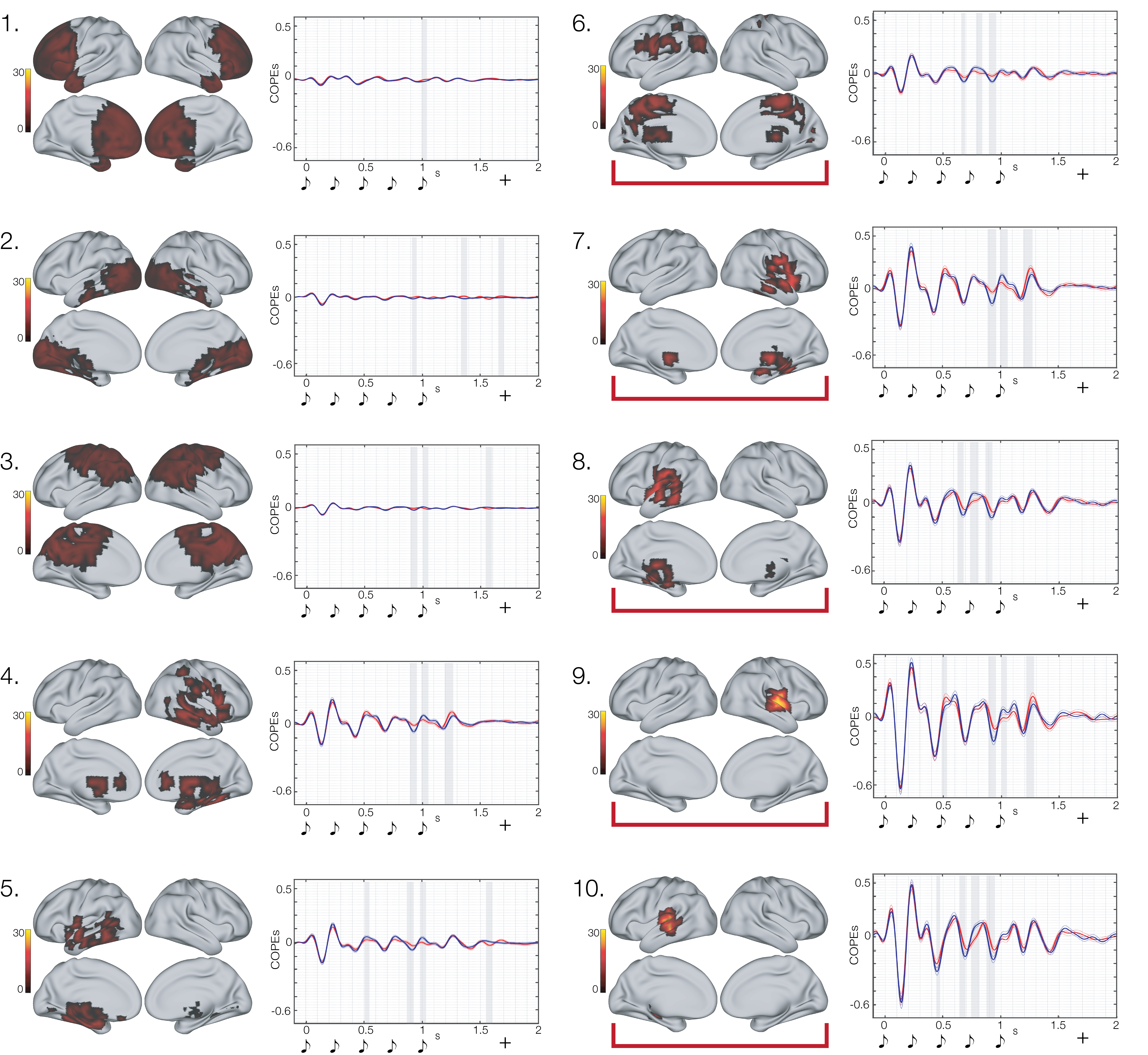

Supplement: FigureS10_bhac439 [file figures10_bhac439.zip › FigureS10_bhac439.tiff]
