# Supplementary material for: Brain recognition of previously learned versus novel temporal sequences: a differential simultaneous processing
Source: Cereb Cortex. 2022 Nov 8;33(9):5524–37. doi: 10.1093/cercor/bhac439 (PMC10152090; doi:10.1093/cercor/bhac439)
Supplement: SupplementaryMaterial_bhac439 [file supplementarymaterial_bhac439.docx]

*Supplementary material for*

Brain recognition of previously learned versus novel temporal sequences: a differential simultaneous processing

L. Bonetti^1,2,3,,5^*, E. Brattico^1,4^, S.E.P. Bruzzone^8,9,1^, G. Donati^5^, G. Deco^6^, D. Pantazis^7^, P. Vuust^1^, M.L. Kringelbach^1,2,3^

*^1^Center for Music in the Brain, Department of Clinical Medicine, Aarhus University & The Royal Academy of Music Aarhus/Aalborg, Denmark*

*^2^Centre for Eudaimonia and Human Flourishing, Linacre College, University of Oxford, UK*

*^3^Department of Psychiatry, University of Oxford, Oxford, United Kingdom*

*^4^Department of Education, Psychology, Communication, University of Bari Aldo Moro, Italy*

*^5^Department of Psychology, University of Bologna, Italy*

*^6^Computational and Theoretical Neuroscience Group, Center for Brain and Cognition, Universitat Pompeu Fabra, Barcelona, Spain*

*^7^McGovern Institute for Brain Research, Massachusetts Institute of Technology (MIT), Cambridge, USA*

*^8^Neurobiology Research Unit (NRU), Copenhagen University Hospital Rigshospitalet, Copenhagen, Denmark*

*^9^Faculty of Health and Medical Sciences, University of Copenhagen, Copenhagen, Denmark*

**Correspondence to: leonardo.bonetti@psych.ox.ac.uk*

***SUPPLEMENTARY RESULTS***

**Modelling the brain activity underlying temporal sequence recognition**

Once the difference between M and N was proved and described in detail, we focused on a further aim of the study, which was to mathematically characterize the dual simultaneous brain processing happening during recognition of the previously memorized temporal sequences. Thus, we computed another round of k-means functional clustering. This time, such analysis was performed only on the group-level main effect of M, to outline a functional parcellation based on the sole recognition of previously memorized sequences. As shown in **Fig. S11 and S12**, the algorithm returned similar results compared to the previous round of k-means functional clustering, but better highlighted the parcels comprising brain areas implicated in memory and evaluative processes.

With regards to modelling, we hypothesized two different mathematical equations (one for each frequency band) that could describe the brain activity over our functionally defined parcels for global and local brain processes.

Regarding the slower frequency band (global processing of the pattern), we used a simple Gaussian function, described as follows (1):

|  | $f(x)=ae^{\frac{{(x-x_{0})}^{2}}{2\sigma^{2}}}$ | (1) |
| --- | --- | --- |

where *a* modulates the amplitude of the curve, *x_0_* shifts it over time and *σ* determines its width.

This equation was fitted using a widely adopted non-linear least square approach, whose results are reported in **Table S9** and depicted in **Fig. S11**. This procedure returned rather good results, highlighting key similarities and differences between the parcels timeseries. The main functional parcels returned a similar peak amplitude (*a*). Conversely, the latencies of such peaks were highly different and shifted over time, as illustrated by parameter *x_0_*. Further, the width of the Gaussian function (indexed by *σ*) varied over the parcels. Indeed, lower-level brain areas such as right and left auditory cortices presented a reduced width compared to higher-level brain areas such as cingulate, insula, hippocampus, inferior temporal cortex and frontal operculum. This result may suggest that the transition from low- to high-order brain areas at the basis of the global processing of the temporal sequence, is also reflected in a longer computation of the information operated by the brain.

Conversely, with regards to the 2-8Hz band (local processing of the items forming the sequence), we hypothesized the following equation (2):

|  | $f(x)=\sum_{i=1}^{N} a_{i}e^{\frac{{(log(x)-x_{0i})}^{2}}{2{\sigma_{i}}^{2}}}*cos(cx+\varphi)$ | (2) |
| --- | --- | --- |

where *a*, *x_0_*_,_ *σ* describes a Gaussian function, exactly as reported for equation (1). This new equation gives rise to a sinusoidal curve that modulates its amplitude based on the associated Gaussian function. As usual, *c* refers to the angular frequency, while *φ* indicates the phase. Finally, *N* refers to the total number of items forming the temporal sequence. This equation was hypothesized since it produces ‘wavelet-like’ timeseries, arguably describing the well-established series of components (peaks of activity in the timeseries, e.g. P50, N100, P300, N300 ^84^) generated by the brain in response to a sound. Indeed, such components have different latencies with respect to the sound onset and present opposite polarities (i.e. P50 and P300 are positive, while N100 and N300 are negative), giving rise to a wavelet-looking timeseries. Although well-established, it is not clear how these components relate to each other, especially when there are multiple brain sources involved and during complex cognitive processes such as recognition of temporal patterns. As done for equation (1), equation (2) was also fitted using the non-linear least square algorithm, returning good results (**Table S9** and **Fig. S12**). However, in this case, the interpretation of the fitted parameters was more complicated since the brain responses to any two subsequent sounds was partially overlapping (i.e. the N300 component enhanced by the first sound occurred with a latency of approximately 320ms and overlapped with the P50 component arising after 50ms from the onset of the second sound). This fact partially altered the contour of the ‘wavelets’ and made the interpretation of the parameters less straightforward. Nevertheless, *x_0_* showed that the centre of the ‘wavelets’ was progressively shifted over time following the onset of the sounds. Moreover, *a* indicated a trend of decreased absolute value over time, coherently with the reduced amplitude of the ‘wavelets’ occurring for the last sounds of the sequence.

**Supplementary information on the k-means functional clustering**

In the following, we provide a few conceptual remarks related to the k-means functional clustering and to the current study that should be highlighted. First, the k-means functional clustering has to be computed on source reconstructed brain data. However, such data can be either the timeseries outputted by the source reconstruction or the timeseries of the statistics computed on the source reconstruction. Moreover, the algorithm can be computed independently for each participant or on the group level statistics. Further, the brain data in input can either be the broadband data or the data reconstructed in selected frequency bands. Moreover, in the likely case of having more than one experimental condition, as conceivable, the algorithm can be computed on each condition independently or on the aggregated (e.g. averaged) conditions. The best procedure cannot be defined a priori for every study and highly depends on the specific aims of the project. In this study, since the main aim of the algorithm was to define functional parcellations with timeseries that best represented the brain functioning among the whole population, we decided to work on the group level statistics. With regards to experimental conditions, we have computed different runs of the k-means functional clustering. Indeed, to statistically compare the timeseries of each parcel for the two experimental conditions of our task, we performed the clustering algorithm on the main effects of the two conditions averaged together. Further, in relation to the frequency bands, we performed one computation of the clustering algorithm for each of the two main frequency bands involved in our study. In the case of 0.1-1 Hz, we worked with absolute values of the reconstructed brain sources timeseries since they did not present any complete cycle of the oscillation, considering their absolute strength as sufficient. Conversely, when dealing with 2-8 Hz, the timeseries presented several complete oscillations and thus computing their absolute values would lead to lose important information. Thus, we resolved the sign ambiguity introduced by the source reconstruction by referencing the sign of the timeseries to the well-known negative polarity of the N100 emerged in response to the first tone of the pattern. Then, we computed the statistics and the subsequent k-means functional clustering on the timeseries which maintained their original double polarity.

***CAPTIONS OF THE SUPPLEMENTARY FIGURES***

*Fig. S1. Temporal, musical sequences used in the experiment.*

*Depiction of all musical sequences employed in the auditory ‘old/new’ paradigm used in the study. On the left, we have shown the 40 musical sequences extracted from the Bach’s prelude (previously memorized musical sequences, ‘old’). On the right, we have shown the 40 novel melodies (novel musical sequences, ‘new’) that were composed and matched to the Bach’s prelude excerpts with regards to several variables such as IC, H and main acoustic features.*

*Fig. S2. MEG sensors waveform and power spectra*

*(****A****) Significantly different brain activity during recognition of ‘memorized’ vs ‘novel’ temporal sequences. The waveforms represent the average over the combined planar gradiometers forming the significant cluster emerged from the analysis, while the grey area illustrates the temporal extent of such significant difference. (****B****) Power spectra computed by using complex Morlet wavelet transform on all MEG channels. The two plots illustrate the power spectra computed for progressively narrower bands. Together with the waveforms, these plots highlight the main contribution of two frequency bands, approximated to 0.1-1 Hz and 2-8 Hz and of a weaker yet distinguishable third band: 8-12 Hz.*

*Fig. S3. Brain activity underlying the single items of the temporal sequences.*

*Significant clusters of brain activity reconstructed in the time-windows corresponding to the five items of the temporal sequences (as illustrated in the first row by the red tones). The brain activity shows the main effects for our experimental conditions (‘memorized’ and ‘novel’) and frequency bands (0.1-1Hz and 2-8Hz). The colorbars indicate one-sample t-values computed for each spatial location and time-point and then corrected with cluster-based permutation tests.*

***Fig. S4. Single-item contrasts in 8-12 Hz band.*** *Contrasts revealed a few, scattered significant clusters where M and N were different, in relation to the five musical tones forming the sequence. The depicted values are t-values obtained by contrasting the brain activity of M versus N in the 8-12 Hz band.*

*Fig. S5. Brain activity underlying recognition of temporal sequences (0.1-1 Hz, single-item) – baselines comparison*

*Brain sources activity in 0.1-1 Hz underlying recognition of each item (musical tone) of the temporal sequences. Results are reported for recognition of ‘memorized’ (M) versus ‘novel’ (N) sequences. The four rows of brains depict the results obtained using the original baseline (100 ms) and the results returned by three additional analyses computed with three different baselines (500 ms, 1000 ms, and 2000 ms). The depicted values are t-values obtained by contrasting the brain activity of M versus N in the 0.1-1 Hz band.*

*Fig. S6. Description of the k-means functional clustering (A) The brain activity is recorded during the recognition of temporal sequences. Such activity, reconstructed in 3559 brain sources, is the input for the k-means functional clustering to define a discrete functionally based parcellation. (B) A functional k-means clustering is performed. Such procedure consists of computing a series of k k-means clustering solutions (e.g. from k = 2 to k = 20) on the functional profile of the brain sources timeseries. In our study, we proposed two simple functional features: the time-index of the peak activity or the actual peak activity value of each brain source timeseries. The example reported in the figure shows clustering on time-indices of peak activity. The first plot shows the heuristic named elbow rule which helps to define the best k solution by plotting the sequential sum of squared errors (SSE) of the different cluster solutions (with k = 2:20). Here, it is visible how the SSE reduces its change rate around k = 6 (as indicated by the circle). Notably, when computing k-means clustering on randomized data, the SSE is higher for randomized vs real data, especially around k = 6, suggesting that the real data should be indeed clustered in six different clusters. As an alternative, the subsequent plot shows the Silhouette value for each k, representing how well each element (brain source time-index) is representative of the cluster to which it belongs. Ideally, those two heuristics should be considered together to define the best k. The plot on the right shows the brain source peak value indices in a violin-scatter fashion, while the plot below provides the same information with time on the x-axis and different colors for the six identified clusters to increase readability. (C) Brain representation of functional k-means clustering results (functional brain parcels). (D) A spatial k-means clustering is performed on each of the functional brain parcels to define a final parcellation considering both brain functional and spatial information. This procedure uses k-means clustering on the spatial coordinates of each of the brain sources belonging to each functional parcel (as shown especially by the plot of the elbow rule for all the six functional parcels). Then, to provide a specific example, the figure focuses on the third functional parcel (indicated by the red brace), whose plots for Silhouette heuristics are reported. (E) Graphical depiction of spatial parcels computation (bottom plot) for the third functional parcel (top plot). (F) Example of few final ‘k-means functional’ parcels and corresponding timeseries, obtained by averaging the timeseries of each brain source belonging to the parcel.*

*Fig. S7. Functional parcellation for 0.1-1 Hz frequency band*

*Full parcellation returned by the k-means functional clustering computed on the indices of the brain activity peaks of all 3559 brain reconstructed sources. This parcellation was computed for the brain activity underlying recognition of memorized temporal sequences. The red brackets show the parcels that are reported in* ***Fig. 3.***

*Fig. S8. Functional parcellation for 2-8 Hz frequency band*

*Full parcellation returned by the k-means functional clustering computed on the brain activity peak values of the timeseries of all 3559 brain reconstructed sources. This parcellation was computed for the brain activity underlying recognition of memorized temporal sequences. The red brackets show the parcels that are reported in* ***Fig. 3.***

*Fig. S9. Full-parcellation contrasts between ‘memorized’ vs ‘novel’ temporal sequences in 0.1-1 Hz*

*Full parcellation and corresponding timeseries returned by the k-means functional clustering computed on the indices of the brain activity peaks of all 3559 brain reconstructed sources. In this case, the parcellation was computed for the averaged brain activity underlying recognition of ‘memorized’ and ‘novel’ temporal sequences. The brain parcels are numbered progressively with decreasing size (i.e. number of brain sources belonging to each parcel). The graphical depiction of musical tones indicates the onset of the items forming the temporal sequence, while the ‘+’ shows the man reaction time of participants’ response. Grey areas illustrate the significantly different time-windows between M and N. In the waveform plots, the solid line corresponds to the mean brain activity, while the dash line to the correspondent standard errors. The red brackets show the parcels that are reported in* ***Fig. 3****.*

*Fig. S10. Full-parcellation contrasts between ‘novel’ vs ‘memorized’ temporal sequences in 2-8 Hz*

*Full parcellation and corresponding timeseries returned by the k-means functional clustering computed on the brain activity peak values of the timeseries of all 3559 brain reconstructed sources. In this case, the parcellation was computed for the averaged brain activity underlying recognition of ‘memorized’ and ‘novel’ temporal sequences. The brain parcels are numbered progressively with decreasing size (i.e. number of brain sources belonging to each parcel). The graphical depiction of musical tones indicates the onset of the items forming the temporal sequence, while the ‘+’ shows the man reaction time of participants’ response. Grey areas illustrate the significantly different time-windows between N and M. In the waveform plots, the solid line corresponds to the mean brain activity, while the dash line to the correspondent standard errors. The red brackets show the parcels that are reported in* ***Fig. 3****.*

*Fig. S11. Full-parcellation fitting for ‘memorized’ temporal sequences in 0.1-1 Hz*

*All parcels whose timeseries were describable by a Gaussian function are reported in this figure. In a few cases, it was not possible to fit the equations since the timeseries showed a very small and scattered activity over time. This happened when those brain areas were not involved in the experimental task. For instance, this was the case of a large occipital parcel that, as conceivable, did not play any role in recognition of auditory sequences. The depicted parcels and corresponding timeseries were returned by the k-means functional clustering computed on the indices of the brain activity peaks of all 3559 brain reconstructed sources. This parcellation was computed for the brain activity underlying recognition of memorized temporal sequences (see Methods for details). The brain parcels are numbered progressively with decreasing size (i.e. number of brain sources belonging to each parcel). The graphical depiction of musical tones indicates the onset of the items forming the temporal sequence, while the ‘+’ shows the man reaction time of participants’ response. In the waveform plots, the solid line corresponds to the actual brain activity, while the dash line to the predicted timeseries obtained by using non-linear least square fitting. The red brackets show the parcels that were mainly relevant for our results.*

*Fig. S12. Full-parcellation fitting for ‘memorized’ and ‘novel’ temporal sequences in 2-8 Hz*

*All parcels whose timeseries were describable by a skewed Gaussian function multiplied by a sinusoidal function are reported in this figure. Only in one case which regarded a large occipital parcel, it was not possible to fit the equation since the timeseries showed a very small and scattered activity over time. This happened since, as conceivable, the occipital cortex did not play any role in the processing and recognition of auditory sequences. The depicted parcels and corresponding timeseries were returned by the k-means functional clustering computed on the brain activity peak values of the timeseries of all 3559 brain reconstructed sources. The two parcellations reported in the figure were computed for the brain activity underlying the recognition of either the ‘memorized’ (left column) or the ‘novel’ temporal sequences (right column) (see Methods for details). The brain parcels are numbered progressively with decreasing size (i.e. number of brain sources belonging to each parcel). The graphical depiction of musical tones indicates the onset of the items forming the temporal sequence, while the ‘+’ shows the man reaction time of participants’ response. In the waveform plots, the solid line corresponds to the actual brain activity, while the dash line to the predicted timeseries obtained by using non-linear least square fitting. The red brackets show the parcels that were mainly relevant for our results.*

***CAPTIONS OF THE SUPPLEMENTARY TABLES***

*Table S1. Brain activity underlying recognition of temporal sequences (single-item)*

*Extensive brain sources activity in the three frequency bands (0.1-1 Hz, 2-8 Hz, and 8-12 Hz) underlying recognition of each item (musical tone) of the temporal sequences. Results are reported for recognition of ‘memorized’ (M) and ‘novel’ (N) sequences independently as well as for their contrasts. Brain areas refer to the automatic anatomic labelling (AAL) parcellation labels, while t indicates the t-value obtained by contrasting M vs N temporal sequences.*

*Table S2. Brain activity underlying recognition of temporal sequences (0.1-1 Hz, single-item) – baselines comparison*

*Extensive brain sources activity in 0.1-1 Hz underlying recognition of each item (musical tone) of the temporal sequences. Results are reported for recognition of ‘memorized’ (M) versus ‘novel’ (N) sequences. The first excel sheet reports the results obtained using the original baseline (100 ms), while the other three sheets show the results returned by three additional analyses computed with three different baselines (500 ms, 1000 ms, and 2000 ms). Brain areas refer to the automatic anatomic labelling (AAL) parcellation labels, while t indicates the t-value obtained by contrasting M vs N temporal sequences.*

*Table S3. Functionally-based parcellation for recognition of ‘memorized’ sequences – 0.1-1 Hz*

*Description of the brain sources belonging to each of the parcels returned by the k-means functional clustering. For each source, the table reports a descriptive label (referring to automatic anatomic labelling (AAL) parcellation), hemisphere, MNI coordinates, and maximum t-value registered in the source timeseries.*

*Table S4. Functionally-based parcellation for recognition of ‘memorized’ and ‘novel’ sequences – 0.1-1 Hz*

*Description of the brain sources belonging to each of the parcels returned by the k-means functional clustering. In this case, the clustering algorithm has been performed on the brain activity averaged over experimental conditions (‘memorized’ and ‘novel’). For each source, the table reports a descriptive label (referring to automatic anatomic labelling (AAL) parcellation), hemisphere, MNI coordinates, and maximum t-value registered in the source timeseries.*

*Table S5. Functionally-based parcellation for recognition of ‘memorized’ sequences – 2-8 Hz*

*Description of the brain sources belonging to each of the parcels returned by the k-means functional clustering performed for ‘memorized’ patterns. For each source, the table reports a descriptive label (referring to automatic anatomic labelling (AAL) parcellation), hemisphere, MNI coordinates, and maximum t-value registered in the source timeseries.*

*Table S6. Functionally-based parcellation for recognition of ‘novel’ sequences – 2-8 Hz*

*Description of the brain sources belonging to each of the parcels returned by the k-means functional clustering performed for ‘novel’ patterns. For each source, the table reports a descriptive label (referring to automatic anatomic labelling (AAL) parcellation), hemisphere, MNI coordinates, and maximum t-value registered in the source timeseries.*

Table S7. Functionally-based parcellation for recognition of ‘memorized’ and ‘novel’ sequences – 2-8 Hz

Description of the brain sources belonging to each of the parcels returned by the k-means functional clustering. In this case, the clustering algorithm has been performed on the brain activity averaged over experimental conditions (‘memorized’ and ‘novel’). For each source, the table reports a descriptive label (referring to automatic anatomic labelling (AAL) parcellation), hemisphere, MNI coordinates, and maximum t-value registered in the source timeseries.

Table S8. Brain activity underlying recognition of temporal sequences (k-means functional clustering)

Contrasts between brain activity underlying recognition of ‘memorized’ vs ‘novel’ temporal sequences. Here, the contrasts have been performed on the timeseries of the parcels returned by the k-means functional clustering computed on the brain activity averaged over experimental conditions. The table provides results for both of our main two frequencies (0.1-1. Hz and 2-8 Hz). Further, for each parcel, it reports size (*k*), *p-value* corrected by Monte-Carlo simulations, temporal extent, and averaged *t-value* of the significant clusters.

Table S9. Fitted coefficients over all parcels’ timeseries (non-linear least square)

R^2^ and coefficients derived from non-linear least square fitting of the equations (5), (6) and (7) reported in the Methods section on the brain activity underlying temporal sequence recognition. In a few cases, it was not possible to fit the equation since the timeseries showed a very small and scattered activity over time. This happened when those brain areas were not involved in the experimental task. For instance, this was the case of a large occipital parcel that, as conceivable, did not play any role in recognition of auditory sequences. The reported parcels were returned by the k-means functional clustering computed either on the indices or on the actual brain activity maximum values of all 3559 brain reconstructed sources. This parcellation was computed for the brain activity underlying recognition of ‘memorized’ temporal sequences for 0.1-1 Hz and ‘novel’ temporal sequences for 2-8 Hz only (see Methods for details).
